# ID 37 - Avaliação Pós-Incorporação do Mesilato de Imatinibe para Tratamento do Tumor do Estroma Gastrointestinal

**DOI:** 10.5327/2237-9622.2025.v34s1.37

**Published:** 2025-11-25

**Authors:** Mirian Carvalho de Souza, Cristiano Guedes Duque, Laura Augusta Barufaldi

**Keywords:** tumores do estroma gastrointestinal, mesilato de imatinib, avaliação da tecnologia biomédica

## Abstract

**Introdução:**

No ano de 2014, em conformidade com a recomendação da Comissão Nacional de Incorporação de Tecnologias no Sistema Único de Saúde (Conitec), o medicamento mesilato de imatinibe foi incorporado ao Sistema Único de Saúde (SUS), como tratamento adjuvante para indivíduos com tumor do estroma gastrointestinal (Gist) que tiveram o sítio primário ressecado. Com o objetivo de manter a sustentabilidade do sistema, é importante que se proceda à avaliação do desempenho das tecnologias incorporadas, com dados de mundo real. O presente estudo teve como objetivo avaliar o desempenho do mesilato de imatinibe para tratamento adjuvante do Gist no âmbito do SUS, desde sua incorporação em 2014.

**Método:**

O estudo foi realizado em três etapas, sendo elas: estudo de revisão sistemática de literatura; estudo observacional com acompanhamento de uma coorte retrospectiva em um hospital de referência para tratamento oncológico no Rio de Janeiro; e avaliação do uso da tecnologia, como tratamento adjuvante para indivíduos com Gist, na rotina do SUS, com base em registros das bases de dados secundários do sistema.

**Resultados:**

O estudo de revisão sistemática de literatura trouxe como acréscimos um novo estudo randomizado fase III e duas revisões sistemáticas, além das atualizações dos outros ensaios clínicos, que, em conjunto, corroboram os dados prévios de eficácia e segurança. O estudo observacional incluiu 41 usuários, sendo que 34,1% deles foram submetidos também a tratamento neoadjuvante com mesilato de imatinibe. O tamanho tumoral era maior que 10 cm em 78,0% dos casos, o que indica alto risco de recidiva, independentemente da localização do tumor primário e do índice mitótico. Com um seguimento mediano de 58 meses, as probabilidades de sobrevida livre de recorrência e de sobrevida global em cinco anos foram estimadas em 64,0% (IC 95%: 43,8% a 78,5%) e de 84,1% (IC 95%: 65,4% a 93,1%), respectivamente. A avaliação das bases de dados do SUS mostrou que a incorporação demorou tempo maior que o previsto para chegar à população brasileira e que o custo do medicamento teve redução significativa desde a sua incorporação.

**Conclusão:**

A avaliação do desempenho do mesilato de imatinibe como tratamento adjuvante do Gist corrobora o relatório n.º 117 da Conitec, que recomendou sua incorporação no SUS para indivíduos com alto risco de recidiva. A atualização da revisão sistemática da literatura reforçou os resultados de eficácia e de segurança publicados do relatório supracitado. Dada a baixa incidência do Gist, a análise de efetividade clínica foi limitada pelo reduzido número de participantes, apesar disso, os achados sobre os desfechos clínicos e a avaliação de segurança foram considerados compatíveis com os obtidos previamente em ensaios clínicos internacionais e multicêntricos. Por fim, a avaliação do uso da tecnologia na rotina do SUS mostrou que houve uma demora, maior do que o esperado, para que o tratamento fosse incorporado de fato e disponibilizado para os indivíduos com Gist. Além disso, a inclusão do mesilato de imatinibe como produto estratégico para o SUS impactou na redução substancial do valor pago pelo medicamento, o que certamente contribui para o favorecimento da relação de custo-efetividade.

